# The application and trend of ultra-weak photon emission in biology and medicine

**DOI:** 10.3389/fchem.2023.1140128

**Published:** 2023-02-17

**Authors:** Jinxin Du, Tingting Deng, Baorui Cao, Zhiying Wang, Meina Yang, Jinxiang Han

**Affiliations:** ^1^ College of Traditional Chinese Medicine, Shandong University of Traditional Chinese Medicine, Jinan, China; ^2^ NHC Key Laboratory of Biotechnology Drugs (Shandong Academy of Medical Sciences), Biomedical Sciences College, Shandong First Medical University, Jinan, China

**Keywords:** ultra-weak bioluminescence, ultra-weak photon emission, reactive oxygen species (ROS), oxidative stress, TCM research

## Abstract

Ultra-weak bioluminescence, also known as ultra-weak photon emission (UPE), is one of the functional characteristics of biological organisms, characterized by specialized, low-energy level luminescence. Researchers have extensively studied UPE for decades, and the mechanisms by which UPE is generated and its properties have been extensively investigated. However, there has been a gradual shift in research focus on UPE in recent years toward exploring its application value. To better understand the application and trend of UPE in biology and medicine, we have conducted a review of relevant articles in recent years. Among the several topics covered in this review is UPE research in biology and medicine (including traditional Chinese medicine), primarily focused on UPE as a promising non-invasive tool for diagnosis and oxidative metabolism monitoring as well as a potential tool for traditional Chinese medicine research.

## 1 Introduction

The luminescence of fireflies is a common biological phenomenon in nature. It is generally accepted that the relaxation of oxyluciferin excited by luciferase enzyme from the excited state to the ground state emits photons and is called bioluminescence (Yeh and Ai, 2019; Adams and Miller, 2020; Wang and Liu, 2021). Only a few species of organisms exhibit bioluminescence due to specific biochemical reactions, such as insects, fish, algae, and fungi (Haddock et al., 2010; Widder, 2010; Al-Handawi et al., 2022). It is, however, important to note that all living organisms (animals, plants, bacteria, fungi, yeasts, humans) continuously emit ultra-weak photons spontaneously without any external stimulation, which is known as ultra-weak bioluminescence, biophoton emission, ultra-weak photon emission, etc. (Niggli, 1992; Devaraj et al., 1997; Vogel and Süssmuth, 1999; Niggli et al., 2003; Tafur et al., 2010; Prasad and Pospisil, 2013).

Gurwitsch (Gurwitsch, 1923) made the first breakthrough in this field in 1923. However, it did not receive much attention at the time due to the poor technology available for detecting photons. The existence of ubiquitous UPE in living organisms was not demonstrated until the development of the photon multiplier tube (PMT) in the 1950s, which increased the sensitivity and detection capabilities of weak radiation in the visible light spectrum significantly. Most of the early progress toward UPE was made by USSR research teams. As early as the 1970s, studies about UPE attracted the attention of scientists worldwide. Several research groups in Japan (Inaba, 1988), Germany (Popp et al., 1981), Poland (Cadenas, 1984), Australia (Quickenden et al., 1985), the USA (18), and China (Yan and Zhang, 1979) have begun to conduct research on the ultra-weak photon emission of biological systems by the use of ultra-low noise, highly sensitive photon counting systems.

Early studies have focused on the fundamentals of UPE, such as the mechanisms and properties of its generation, as well as the factors influencing it. However, the focus of recent studies has shifted to UPE as a potential non-invasive diagnostic tool, the integration of UPE and metabolomics, as well as the relationship between UPE and Traditional Chinese Medicine. The purpose of this review is to provide an overview of the application of UPE in biology and medicine and to discuss some of the future directions of UPE research.

## 2 Characterization of UPE

The intensity of UPE is extremely weak at 10^1^–10^3^photons/sec/cm^2^ (or equivalently 10–16 to 10–18 W/cm^2^), which is weaker by 1/1000 times the sensitivity of the human eye (10–12 to 10–14 W/cm^2^) (Kobayashi, 2003). The ultra-weak intensity characteristic of UPE makes it cannot be detected by the naked eye. However, it can be measured by highly sensitive apparatuses, such as PMT and charge-coupled devices (CCD). And there has been evidence that the spectral range of UPE ranges from the near ultra-violet A region (UVA) to the visible and infrared regions (IR) of the electromagnetic spectrum (Kobayashi et al., 1999; Cifra and Pospisil, 2014).

UPE was not distributed uniformly throughout the human body. Different body sites showed varying levels of UPE intensity, with some areas showing relatively high and low levels. Despite some disparities in UPE between individuals, the overall distribution pattern is analogous; in other words, the upper extremities and head region have a higher intensity of UPE than the torso (Wijk and Wijk, 2005) and a high degree of left-right symmetry (Cohen and Popp, 1997a).

There were factors that could have an impact on UPE. The results of previous research have shown that external factors such as external light (Niggli et al., 2005; Wijk and Wijk, 2005), diurnal rhythm (Author Anonymous et al, 2007; Cifra et al., 2007; Kobayashi et al., 2009), time of the year (Jung et al., 2005; Wijk and Wijk, 2005), and oxygen concentration (Nakamura and Hiramatsu, 2005) have some influence on the emission of UPE. In spite of this, studies have shown that UPE is linked to physiological and pathological states. Some internal factors, such as age (Zhao et al., 2016), gender (Cohen and Popp, 1997b; Zhao et al., 2016), and consciousness activities (Van Wijk et al., 2005; Van Wijk et al., 2008a; Van Wijk et al., 2008b), can also affect the intensity of photon emission.

## 3 UPE and oxidative stress

Reactive oxygen species (ROS) are collections of metabolites derived from molecular oxygen, whose instability results in easy reactions with other molecules in the cell (Dickinson and Chang, 2011). ROS are products of metabolic activities that, at low/medium concentrations, play critical roles in intracellular homeostasis, cell proliferation, cell death, immune defense against pathogens, as well as gene and protein expression. However, excessively high levels of ROS can adversely affect the body (Murphy et al., 2011; Ray et al., 2012; Schieber and Chandel, 2014; Pizzino et al., 2017). The antioxidant system within living organisms has been developed to protect cellular components against oxidative damage caused by ROS. However, in some cases, the formation of ROS exceeds the capacity of the antioxidant system, resulting in oxidative stress. As a result of oxidative stress, multiple organs and systems can be adversely affected, and excess free radicals produced by oxidative stress can damage proteins, lipids, and DNA, leading to the development of diseases such as diabetes, neurodegenerative diseases, cardiovascular diseases, rheumatoid arthritis, and cancer (Dhalla et al., 2000; Newsholme et al., 2016; Prasad et al., 2017; Phull et al., 2018; Sumien et al., 2021). The importance of oxidative stress in physiopathology makes it imperative to find an analytical method that can continuously and non-invasively monitor oxidative metabolic status *in vivo*.

### 3.1 The mechanism of UPE production

**FIGURE 1:**
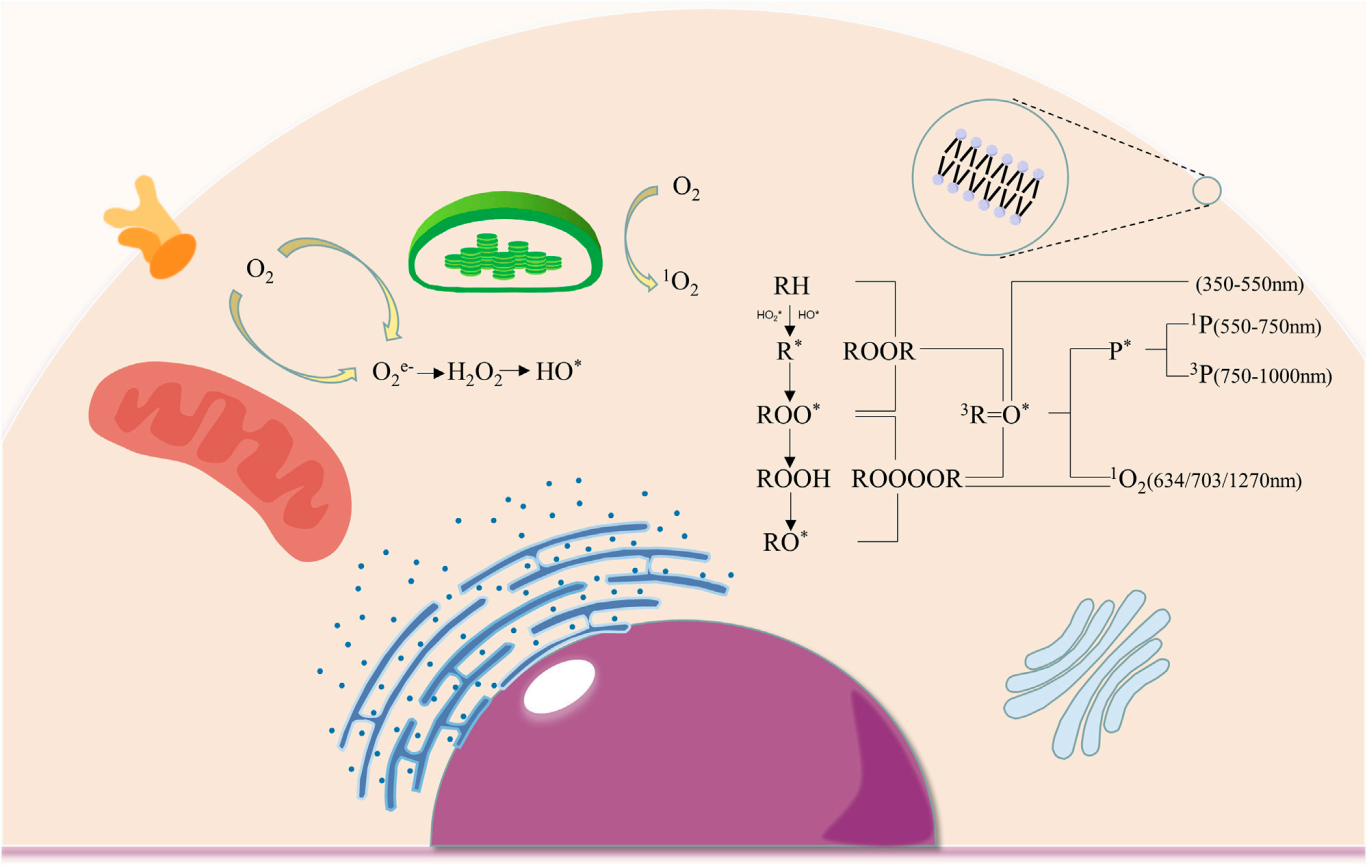
The production of intracellular ROS and mechanisms of electronically excited species formation. (ROS production occurs in cell membranes, chloroplasts and mitochondria, where oxygen was converted into O_2_e-, H_2_O_2_, HO^*^ and 1O_2_; the oxidation of biomolecules by ROS leads to the formation of 3R=O^*^, 1P^*^, 3P^*^ 1O_2_ respectively, resulting in photon emission at different spectral wavelengths.).

The photon emissions of UPE are believed to be primarily attributed to the relaxation of electronically excited species (Figure 1) formed during the oxidative metabolic processes of lipids, proteins, and nucleic acids by reactive oxygen species in biological systems (Pospisil et al., 2019). The oxidative reaction of ROS on biomolecules initiates the decomposition of the unstable high-energy intermediates 1,2-dioxetane and tetroxide, thereby starting the formation of triplet excited carbonyls (^3^R = O^*^). When pigments are in close proximity with excited carbonyl species, the non-radiative energy transfer results in the formation of singlet (^1^P^*^) and triplet (^3^P^*^) excited pigments, whereas when the molecule oxygen is in contact, singlet oxygen (^1^O_2_) is formed (Cifra and Pospisil, 2014; Pospisil et al., 2019). These triplet excited carbonyls, singlet excited pigments, triplet excited pigments, and singlet oxygen contribute to the photon emission in the near UVA and blue-green (350–550 nm) regions, green-red (550–750 nm) region, red-near IR (750–1000 nm) region, red (634 and 703 nm) and near IR (1270 nm) regions of the spectrum respectively (Mano et al., 2014; Prasad et al., 2018). The mitochondria are the predominant source of photon emission by the oxidative phosphorylation in the membrane, which produces the most ROS in cells (Shanei et al., 2017).

### 3.2 UPE as an oxidative metabolism monitor

Considering that UPE is associated with oxygen consumption, ROS formation, and electron excited states, it is theoretically possible to use UPE to monitor the organism’s oxidative metabolism and stress (Tafur et al., 2010). As early as 1980, Boveris et al. (1980)explored the possibility of using chemiluminescence techniques to continuously monitor organ metabolism *in vivo*. After the injection of exogenous hydroperoxides, they observed an increase in UPE by up to 30 times. Adamo et al. (1989) reported that in 1989, hyperthyroid rats induced with tri-iodothyronine suffered hypermetabolism and oxidative stress, resulting in a higher UPE than controls. The results of Kobayashi et al. (1999) showed that oxidative stress induced by hyperoxia (100% oxygen inhalation) enhanced the intensity of UPE in rat brains by approximately 130%, followed by a gradual decrease in photon emission intensity as oxygen concentrations returned to normal levels (45% oxygen inhalation). Studies described above explore the potential application of UPE in the non-invasive monitoring of oxidative metabolism and oxidative stress in living tissues. There also has been some research further demonstrating the feasibility of using UPE as a tool for the dynamic monitoring of oxidative stress at the cellular level. Shanei et al. (2017) found that UPE was significantly elevated in HT-29 cells after the application of H_2_0_2_. The association between UPE and ROS was further explored by using HL-60 cells, which were differentiated into neutrophil-like cells with all-trans retinoic acid and then induced respiratory bursts with phorbol 12-myristate 13-acetate (PMA). A significant increase in UPE was observed after PMA treatment in cells treated with all-trans retinoic acid for 7 days. Furthermore, a significant reduction of UPE was observed after the application of DPI, an inhibitor of NADPH oxidase (Burgos et al., 2017). The review by (Vahalova and Cifra, 2023)presented in detail on how UPE can be used to report on various aspects of the cellular oxidation process as well as the advantages and disadvantages of UPE as a biotechnology in monitoring biological oxidation.

In general, studies on UPE and oxidative stress are mainly in dermatology (Table 1). As the outermost barrier of the body, the skin is exposed to external factors such as UV, oxygen, and pollutants, which leads to increased ROS production. Aside from that, the skin is continuously exposed to endogenous ROS-induced oxidative effects, which means that the skin has a higher ROS load than any other organ in the body (Rinnerthaler et al., 2015). The oxidative stress caused by elevated ROS levels can lead to a reduction in the structural integrity of the skin as well as a loss of physiological function, which increases the risk of skin diseases and skin cancers (Durai et al., 2012; Kammeyer and Luiten, 2015). As a result, monitoring oxidative stress in the skin is of great importance for anti-aging and skin disease prevention. However, most of the modern techniques are based on the detection of markers of oxidative stress in skin samples or biopsies of tissue or blood (Hagens et al., 2008; Alexandrova and Bochev, 2009). There is a lack of tools for non-invasive spatiotemporal monitoring of skin oxidative metabolism.

**TABLE 1:** Studies on the association between UPE and skin disorders.

Subjects	Measurement components	Body parts	Main results/conclusions	Year	Reference
Porcine skin samples *in vitro*	PMT	—	UPE generated by oxidation-stressed skin is mainly due to non-fluorescent photon emission *via* Trp amino acid; Measurement of UPE could be a highly sensitive method to assess oxidative processes in biological molecules	2007	Khabiri et al. (2008)
Porcine skin samples *in vitro*; Human skin *in vivo*	PMT	The inner forearms (*in vivo*)	UPE measurement following UVA excitation could precisely reflect a dose-dependent antioxidant effect of topically applied vitamin C and alpha-glucosyl rutin	2007	Hagens et al. (2008)
Human skin *in vivo*	PMT and CCD camera	The dorsal and the palm side of the hand	UPE increased after the application of H_2_O_2_; UPE measurement serves as a powerful non-invasive tool for monitoring peroxide-induced oxidative processes in the human skin	2010	Rastogi and Pospisil (2010)
Human skin *in vivo*	PMT	The dorsal surface of the hands	UV exposure resulted in an increase in UPE; The specific topical OPCs cream formulation reduced UV-induced UPE in the skin; The UPE measurement protocol can be utilized for the routine evaluation of the antioxidant efficacy of topical formulations on human skin	2010	Van Wijk et al. (2010)
Human skin *in vivo*	PMT and CCD camera	The dorsal side of the right hand	Spontaneous UPE can be used as a non-invasive tool for the temporal and spatial monitoring of the oxidative metabolic processes and intrinsic antioxidant system in human skin	2011	Rastogi and Pospisil (2011)
Human skin *in vivo*	PMT and CCD camera	The dorsal and palmar sides of the hand	Both UVA radiation and visible light exposure led to increased UPE; Two-dimensional photon imaging can serve as a potential tool for monitoring the oxidative stress in the human skin induced by various stress factors irrespective of its physical or chemical	2012	Prasad and Pospisil (2012)
Human skin *in vivo*	PMT	The inner upper arm and the outer forearm for women; the inner upper arm, outer forearm, and buttock for men	Steady-state UPE reflects not only intrinsic skin aging and cutaneous color but also the current oxidative status independent of skin aging	2014	Gabe et al. (2014)
Human skin tissue *in vitro*; Human skin *in vivo*	CCD camera	The backs of the Fingers (*in vivo*)	UPE measurement is a useful method to evaluate UV-induced oxidation in the human skin, and UPE imaging is an effective method to visually evaluate oxidative stress in the human skin	2019	Tsuchida et al. (2019b)
Human skin *in vivo*	CCD camera	The facial skin	Upe intensity was correlated with porphyrin score in the skin; UPE imaging of facial skin revealed regional variations of oxidative stress and site-specific increases in oxidative stress with age	2020	Tsuchida and Kobayashi (2020)
Human skin *in vivo*; Normal human epidermal keratinocytes *in vitro*	PMT	The inner upper arm	Long-lasting UPE generated between 1 and 3 min immediately after UV exposure, which is associated with lipid hydroperoxide production, is a valuable indicator to estimate and/or avoid severe cutaneous photodamage	2020	Gabe et al. (2021)

Abbreviations: PMT, photon multiplier tube; CCD, charge-coupled devices; UPE, ultra-weak photon emission.

Several studies have demonstrated that UPE can serve as a non-invasive tool for the dynamic monitoring of oxidative stress in the skin (Hagens et al., 2008; Khabiri et al., 2008; Rastogi and Pospisil, 2011; Gabe et al., 2014; Tsuchida et al., 2019a; Tsuchida and Kobayashi, 2020). According to Hagens et al. (2008), skin UPE emission showed an elevated trend after UVA exposure, while the topical application of vitamin C and alpha-glucosyl rutin significantly reduced it and exhibited a dose-dependent effect on UPE emission. In addition, UPE has also been found to have a significant correlation with skin aging (Gabe et al., 2014).

The above studies are skin UPE studies based on a photomultiplier tube, which provides information such as radiation intensity, while UPE detection by CCD camera technology can provide spatial and temporal information about skin UPE. In the study of (Rastogi and Pospisil, 2011), it was found that skin spontaneous UPE is reduced when anaerobic conditions are present, while it is enhanced when hyperoxic conditions are present, with reduced photon emission after topical application of glutathione, α-tocopherol, ascorbic acid, and coenzyme Q10. Tsuchida et al. (2019a) found that UVA-induced UPE intensity increased in a dose-dependent manner, that sodium l-ascorbate, l-glutathione, and d-δ-tocopherol significantly inhibited the UVA-induced UPE, and that the antioxidant effect of sunscreens was confirmed by CCD camera imaging. Furthermore, there is a study that found that facial wrinkle scores were correlated with the intensity of UPE in the skin (Tsuchida and Kobayashi, 2020). Based on the studies presented above, it can be concluded that UPE detection technology can be used as a non-invasive tool to measure oxidative metabolism and oxidative stress in the skin at a temporal and spatial scale.

## 4 UPE and disease

As mentioned above, UPE production can be affected by internal factors such as age, gender, and brain activity (Cohen and Popp, 1997b; Van Wijk et al., 2005; Van Wijk et al., 2008a; Van Wijk et al., 2008b; Zhao et al., 2016). It should be noted, however, that disease factors can also result in significant changes in UPE parameters (Ives et al., 2014). It was speculated that the UPE measurement could also be used as a non-invasive diagnostic tool due to the strong association between ROS production and the onset and progression of several disorders, including diabetes, neurodegenerative diseases, cardiovascular diseases, rheumatoid arthritis, and cancer (Dhalla et al., 2000; Newsholme et al., 2016; Prasad et al., 2017; Phull et al., 2018; Sumien et al., 2021). A significant portion of the research conducted in recent years has focused on investigating the relationship between UPE and disorders, as well as the feasibility of using UPE as a non-invasive diagnostic tool.

Among the diseases studied, metabolic disorders account for a significant percentage (Table 2). The blood UPE intensity of diabetic patients was found to be 3–4 times higher than that of healthy subjects, while the blood UPE intensity of hyperlipidemic patients was also significantly greater than that of healthy individuals (Inaba et al., 1982). On the other hand, studies in recent years have focused on choosing different body parts for non-invasive UPE testing. Yang et al. (Yang et al., 2017) performed UPE testing on a total of five sites of the human body: forehead, throat, heart, abdomen, and navel in a total of 50 patients with type 2 diabetes using a movable whole-body biophoton detecting system. They found that the UPE emission intensity at the navel was significantly higher in type 2 diabetic patients than in the healthy group. In contrast, the photon intensity at the forehead was significantly lower than in the healthy group. Other UPE parameters such as Q value, compression state parameter, and compression state index were also observed to be different between T2DM patients and healthy individuals. Furthermore, other studies have examined the association between UPE of hands and different “syndromes” of diabetes in Chinese medicine in order to investigate the feasibility of using UPE or combined plasma metabolomics to diagnose different traditional Chinese “syndromes” in diabetic patients (Sun et al., 2017; He et al., 2019).

**TABLE 2:** Studies on the association between UPE and metabolic disorders.

Disease	Subjects	Samples/Measurement site	Main results	Year	Reference
Diabetes	Human	Blood samples from diabetic patients and healthy subjects	Patients with diabetes mellitus showed 3–4 times higher emission levels than healthy control samples; Glucose levels in diabetic subjects were not directly related to emission intensity	1982	Inaba et al. (1982)
Diabetes	Human	Forehead, throat, heart, abdomen, and navel of type 2 diabetic patients and healthy subjects	Patients with diabetes have significantly higher and lower photon intensity in their navel and forehead, respectively than healthy subjects; For the throat and forehead, the Q value and the percentage of signals yielding normal values for squeezed state parameters as well as SSI in type 2 diabetes patients are lower than in healthy subjects	2017	Yang et al. (2017)
Diabetes	Huaman	The dorsal and palm of both hands in pre-diabetic subjects	Out of the 40 parameters obtained, 16 were able to differentiate between the three subtypes of pre-diabetes	2016	Sun et al. (2017)
Diabetes	Human	The dorsal and palm of both hands in pre-diabetic subjects	The three TCM-based subtypes of early-stage type 2 diabetes can be distinguished by plasma metabolomics.	2019	He et al. (2019)
			A correlation was found between UPE parameters and plasma metabolites, primarily lipids, and these correlations differed among the subtypes		
Hyperlipidemic	Human	Blood samples from hyperlipidemia patients and healthy subjects	Blood photon counts were generally higher in subjects with hyperlipidemia	1982	Inaba et al. (1982)

Abbreviations: UPE, ultra-weak photon emission; SSI, squeezed state index; TCM, traditional Chinese medicine.

It also has been found that the blood UPE photon count is three to four times higher in cancer patients than in healthy individuals in early studies (Inaba et al., 1982). Subsequent studies regarding the association between UPE and cancer have been conducted with tissues or animal models (Table 3). There has not been any UPE-related study that could be retrieved using cancer patients as research subjects. As reported by Amano et al. (1995), the number of UPE photons in tumor sites of bladder cancer mice was significantly higher than in normal tissues. Kim et al. (2005) measured the UPE emitted from cancerous frozen tissues and found that the UPE of cancer tissues differed from that of normal tissues, with UPE emission varying for different types of tumors. It was found that the UPE intensity of hepatocellular carcinoma, intermediately differentiated adenocarcinoma, lung cancer, and esophageal cancer tissues was higher than that of normal tissues; the mean UPE intensity of papillary microcarcinoma tissues was slightly higher than that of normal tissues, while the UPE intensity of breast cancer tissues was lower. Takeda et al. (2004) implanted AH109A, TE4, and TE9 cell lines into mice and used a CCD camera system to analyze changes in the UPE of animals at different intervals following tumor cell transplantation. They indicated that the intensity of UPE reflected the viability of tumor tissue and revealed that the biophoton intensity of tumors was positively correlated with tumor size at 1 week (correlation coefficient 0.73). Zhao et al. (2017a) demonstrated the changes in UPE parameters during tumor growth in nude mouse models inoculated with human breast cancer cells. They selected the following parameters as their main criteria: UPE intensity on the left and right sides of the body surface and the ratio of left to right intensity. A significant difference in UPE parameters between the model group and the control group has been observed, and the UPE parameters clearly distinguished tumor-bearing mice from healthy mice, even in the early stages of tumor development. Murugan et al. (2020) also demonstrated that tumor cells emit more photons than non-malignant cells. The results of these studies indicate that UPE is a potential non-invasive tumor screening method, at least *in vitro*. It may be necessary to conduct *in-vivo* studies on cancer patients in order to clarify the feasibility of using UPE as a non-invasive diagnostic tool for detecting cancer. It would be valuable to explore the role of UPE measurements in the early detection of cancer.

**TABLE 3:** Studies on the association between UPE and cancer.

Subjects	Cancer type/Cell line	Detection system components	Reference
Human blood samples	Cancers of the hepatobiliary system and gastrointestinal tract	PMT	Inaba et al. (1982)
Human cancer tissues	Moderately differentiated adenocarcinoma, lung tumor, hepatocellular carcinoma, papillary microcarcinoma, esophagus tumor, breast tumor, leiomyosarcoma, follicular adenoma, infiltrating ductal carcinoma	PMT	Kim et al. (2005)
Mice	Transplanted bladder cancer/KK-47 cells	video-intensified system	Amano et al. (1995)
Mice	Human esophageal carcinoma cell lines: TE4, TE9 and rat hepatoma cell line AH109A	CCD camera	Takeda et al. (2004)
Mice	Breast cancer/MDA-MB-231	PMT	Zhao et al. (2017a)
Mice	AsPC-1, Capan-1, CFPAC-1, B16-BL6, BxPC3, HBL 100, HEK 293, HELA, HPAF-11, HSG, Hs 578T, MCF7, and MDA MB 231	PMT	Murugan et al. (2020)
Mice	Ovarian cancer/OVCAR-3	PMT	Kim et al. (2006)

Abbreviations: PMT, photon multiplier tube; CCD, charge-coupled devices.

Furthermore, several studies have addressed the correlation between UPE and other disorders such as cold (Yang et al., 2015), multiple sclerosis (Cohen and Popp, 1997a), hemiparesis (Jung et al., 2003), rheumatoid arthritis (He et al., 2017), and transplanted ovarian tumors (Kim et al., 2006). However, the number of these studies is relatively small and the indicators used for observation are relatively homogeneous. Further investigation of the application of UPE as a non-invasive diagnostic tool will need to be conducted in future studies that combine UPE parameters with clinical symptoms, laboratory indicators, and Omics analysis.

## 5 UPE and Traditional Chinese Medicine

Traditional Chinese medicine (TCM) has thousands of years of application history and differs significantly from modern medicine in its understanding of diseases and its approach to both the prevention and treatment of illness. TCM has always followed the guiding principle of the “theory that man is an integral part of nature,” which holds that the human body is an open system constantly exchanging information and energy with the external environment (Wang, 2012). TCM has a specific and holistic approach to the management of health, taking the connection and unity between the human body and the environment as its main guiding principle, utilizing acupuncture and herbal medicine, as well as modifying patients’ lifestyles to prevent and treat diseases (Li and Xu, 2011; Dou et al., 2021). The diagnostic and therapeutic approaches of traditional Chinese medicine differ greatly from those of modern medicine; thus, combining the knowledge of Chinese medicine with modern medicine will undoubtedly enhance the development of the medical field (Schroen et al., 2014). However, like other traditional medicine, TCM also faces several significant challenges. It is difficult for scientists with other cultural backgrounds to comprehend complex TCM theories due to the significant differences between TCM theories and modern medicine. In addition, the inadequacy of modern scientific research has limited the dissemination of TCM theories throughout the world. Most TCM research still follows segmentation and reductionist approaches, which are not suited to reflect the holistic and dynamic characteristics of TCM systems. As a result, researchers have devoted considerable time and effort to finding tools and methods that can better reflect TCM’s holistic and dynamic characteristics. One of these methods is the use of UPE measurement.

### 5.1 UPE in meridian and acupuncture research

In TCM, meridians are considered to be an essential part of the body structure, being the main channels for the flow of Qi and blood as well as for the transmission of information, while acupuncture points are points with therapeutic effects distributed throughout the body (Ifrim Chen et al., 2019). In Chinese medicine, the meridian system plays a significant role in understanding pathological changes in disease, as well as guiding the diagnosis and treatment of illness (Wang et al., 2010). Previous studies indicated that UPE measurement is a potential tool for meridian and acupuncture research. It was demonstrated by Yan et al. that the surface of the human body has 14 high-incidence rays, which are highly coincident with the 14 meridian routes (Yan et al., 1989). In addition, the luminescence of the acupuncture points of the 12 main meridians was higher than that of the non-acupuncture points (Yan et al., 1984). A significant change in luminescence intensity was also observed at the distal end of the Jing-Well Points associated with the acupuncture points after the needling of Qi (Yan et al., 1993). A significant difference was found in UPE between the left and right hands of patients with hemiparesis, with a left-right asymmetry characterized by a low UPE on the paralyzed side. However, the asymmetry was significantly improved after acupuncture treatment (Jung et al., 2003). Park et al. (2009) have also found that subjects showed significant changes in photon emission in the palm after magnetic needle stimulation. There is clearly some overlap between these UPE studies and the meridian theory of TCM. It would be a significant discovery if the existence and connotation of the meridian system could be revealed and verified by means of UPE measurement.

### 5.2 UPE and TCM syndrome

“Syndrome differentiation and treatment” is another characteristic of TCM theory. In Chinese medicine, a syndrome is a generalization of a specific stage or type of pathology in the disease process, reflecting the stage-specific nature of the disease (Guo et al., 2006). The same disease can have several different TCM syndromes, while different diseases can also have the same syndrome. “Syndrome differentiation and treatment” is a comprehensive analysis based on the information obtained from the four diagnosis methods of TCM (inspection, listening and smelling examination, inquiry, and palpation) to clarify the essence of the illness and to determine the treatment principles and prescriptions (Jiang et al., 2012). The study of TCM syndromes plays an imperative role in TCM research. In recent years, researchers have been searching for methods that can better match the holistic and dynamic nature of TCM syndromes, while UPE is able to do so precisely. According to Yan et al. (1982), the intensity of UPE on the body surface of animal models of deficiency syndrome was significantly reduced, which varied with the degree of weakness. It was concluded that UPE parameters could serve as an indicator of an organism’s deficiency syndrome status. Sun et al. (2017) performed UPE measurements on 44 pre-diabetic subjects on the palm and dorsum of both hands, while the TCM syndrome of these patients was determined by experienced Chinese medicine practitioners. A total of 40 parameters were obtained for each subject. The subjects were categorized into three TCM syndromes: Qi-Yin deficiency, Qi-Yin deficiency with dampness, and Qi-Yin deficiency with stagnation. Based on statistical analysis, 16 UPE parameters were able to differentiate between the three TCM symptoms in these subjects, with a prediction accuracy of 97.81%. In a subsequent study by the same team, metabolomics and UPE were combined to further analyze these three pre-diabetic TCM syndromes. Their study showed that both plasma metabolomics and UPE parameters were able to distinguish these three subtypes. A correlation analysis revealed that UPE was associated with specific plasma metabolites, primarily lipids (He et al., 2019). In the study conducted by Wang et al. (2020), UPE intensity at the Dazhui acupoint was significantly higher in patients with Spleen-Qi deficiency syndrome than in healthy individuals. It was observed that after the Ginseng treatment was applied, the elevated UPE was significantly reduced. A novel approach to the study of TCM syndromes was proposed based on the results of these studies. UPE has shown to be an extremely promising research tool in the field of TCM syndromes. However, additional explorations and studies are needed to further illustrate the feasibility and accuracy of characterizing TCM syndromes using UPE parameters.

### 5.3 UPE and herbal medicine

Chinese herbal medicine has a thousands-year history of application and plays a critical role in TCM. In the course of applying Chinese herbal medicine for thousands of years, a series of unique theories have been developed. It has been widely recognized in recent years that Chinese herbal medicine can be used as a complementary or alternative therapy for a variety of illnesses (Chaudhury, 2015). Meanwhile, researchers are constantly exploring methods to interpret the theory of herbal medicine using modern scientific techniques and tools, which include UPE and delayed luminescence (DL)—a continuously decaying ultra-weak luminescence from objects that have been exposed to light (Strehler and Arnold, 1951; Popp and Yan, 2002; Scordino et al., 2014).

### A Research on the antioxidant properties of Chinese medicine

As research on TCM progresses, the pharmacological mechanisms by which it exerts its therapeutic effects are being revealed. The antioxidant properties of TCM have been demonstrated to be closely related to some of its effects. A growing awareness of the benefits of natural antioxidants has led to a rising interest in the antioxidant properties of herbal medicines (Benzie and Wachtel-Galor, 2011; Liu and Jiang, 2012; Phu et al., 2020). It is also possible to evaluate the antioxidant properties of herbal medicines using UPE since it can be used as a tool for assessing dynamic oxidative metabolism. Several research groups have also conducted antioxidant studies using UPE on herbal medicines. A randomized controlled trial explored changes in UPE parameters in subjects after the application of Rhodiola Rosea. One week after Rhodiola Rosea administration, the subjects in the Rhodiola Rosea group showed a significant reduction in dorsal photon emission from both hands compared to the placebo group, as well as a statistically significant reduction in the experienced levels of stress and of fatigue (tiredness) (Schutgens et al., 2009). According to another study, Ginseng was also capable of reducing UPE intensity in mice with Spleen Qi deficiency syndrome, which may be attributed to Ginseng’s inhibitory effects on oxidative stress (Wang et al., 2020).

### B Research on the hot and cold properties of Chinese medicine

The hot and cold properties of herbal medicine are important parts of TCM theory, which is of great importance for clinical practice and is also the focus of current research on Chinese medicine (Zhao et al., 2011). It was suggested by Pang et al. (Pang et al., 2016a) that DL, combined with statistical analysis, could provide new methods and parameters for the study of the cold and hot properties of herbal medicines. The subsequent spectral analysis revealed significant differences between cold and hot herbal medicines in terms of spectral distribution and decay probability distribution. The results of this study provided a basis for analyzing the cold and hot properties of DL (Pang et al., 2016b). Scenedesmus obliquus was used as a bioindicator in another study by the same group. Cold and hot herbal decoctions were added to scenedesmus obliquus, respectively, and DL was detected. Based on an elaborate analysis of the parameters, the K value was found to be a useful parameter for distinguishing between the cold and hot properties of herbal medicines (Yang et al., 2022). It appears that the detection of scenedesmus obliquus DL values after drug administration is a promising approach for studying cold and hot medicinal properties. Zhou et al. (Zhou et al., 2019), on the other hand, investigated the UPE in the abdomen and back of mice given cold and hot Chinese herbs by gavage, respectively. Their results showed that the UPE intensity ratios in the abdomen and dorsum were significantly lower in mice treated with cold herbal medicines than in normal controls. In contrast, hot herbal medicines showed the opposite results. All of these findings suggest that both UPE and DL are very promising tools for studying the medicinal properties of herbal medicines.

### C Research on quality control of Chinese medicine

Chinese herbal medicines hold a very prominent position in the clinical practice of TCM. It should be noted, however, that the quality of Chinese herbal medicines varies due to the wide range of sources and origins. Therefore, quality control of Chinese herbal medicines is a major concern in the field. So, if the relationship between UPE or DL parameters and active ingredient content can be established, this may be a promising technique for the quality control of herbal medicines. An analysis of DL results for several types of herbal medicines (Aconite, Rhubarb, and Ginseng) was conducted by Sun et al. (Sun et al., 2016a). It has been shown that DL is a promising tool for assessing the quality of dried herbal medicines and that the combination of DL and chemical analysis provides an effective way to control herbal medicine quality. The same group also investigated the effect of different altitudes on rhubarb chemistry. Rhubarb from different altitudes was collected for HPLC analysis and DL measurements, both of which reflected that the quality of rhubarb was influenced by environmental factors. Spearman correlation analysis revealed a significant correlation between DL and bioactive compounds (Sun et al., 2016b). The study of Zhao et al. (Zhao et al., 2017b) on spontaneous UPE of herbal medicines also showed that UPE parameters could reflect the content of specific active compounds in the same herb in different growth periods. All of these studies suggested that UPE and DL could be potential tools for the quality control of herbal medicines. However, the number of current studies is relatively low, and the variety of herbs tested is limited. Future studies are still needed to conduct a large number of tests in combination with chemical composition analysis.

## 6 Conclusion and prospection

Over the past few decades, UPE has been extensively studied as an inherent function of organisms. It is generally believed that UPE originates from the relaxation of electronically excited species from the excited state to the ground state during oxidative metabolism. As a result, it is widely used in studies that assess oxidative stress *in vivo*, primarily in studies involving the skin. UPE is also a promising non-invasive diagnostic tool that has been investigated by many researchers. However, the number of diseases examined is limited, and large-scale tests are still needed to enrich the basis of UPE as a non-invasive diagnostic tool. Using UPE in conjunction with various omics techniques to explore the modern interpretation of TCM syndromes may be a promising direction of research to assist in explaining the complex TCM syndrome theory. Moreover, combining UPE or DL with chemical analysis for quality control of Chinese herbal medicines would be an excellent research topic. In conclusion, UPE has great potential for application, but further research is required to further verify its reliability.
